# Risk factors for type 2 diabetes mellitus among patients attending a rural Kenyan hospital

**DOI:** 10.4102/phcfm.v2i1.96

**Published:** 2010-05-24

**Authors:** Masemiano P. Chege

**Affiliations:** 1 Department of Family Medicine, Moi University, Kenya

**Keywords:** adult type 2 diabetes mellitus, risk factors, rural population, relative risk, Kenya

## Abstract

**Background:**

The Diabetes Management and Information Center in Nairobi has conducted population surveys among rural and urban Kenyans during the last decade. They have reported a rise in the prevalence of diabetes among rural Kenyans from 3% in 2003 to 7% in 2007. Our study was undertaken to investigate rural factors for type 2 diabetes and determine those that could be responsible for this rise in prevalence.

**Objectives:**

To describe the risk factors for type 2 diabetes mellitus among patients attending the outpatient clinics in a rural mission hospital in Kenya.

**Method:**

Forty-five diabetics and forty-five non-diabetics, resident in this rural hospital's catchment area for at least 10 years, were randomly selected from patients attending outpatient clinics. Diabetics in a stable condition (not requiring hospitalisation), whose fasting blood sugars were below 6.1 mmol/L, were matched for age and gender with the non-diabetics who came to the hospital for outpatient services. A pilot-tested questionnaire on demography, current and past dietary habits, social habits, and family history was used to collect data. Waist circumference, height and weight were measured and BMI calculated. Data was analysed using SPSS for Windows. The Kruskal–Wallis test was used to compare the medians for the continuous variables, while the chi-squared test was used for the categorical variables. The z-test was used to calculate the relative risk.

**Results:**

Ninety participants (26 males, 64 females). The mean age was 61.8 for diabetics and 61.4 for non-diabetics. Childhood starvation (relative risk 2.08, *p* = 0.0090) and use of cassava for sustenance during childhood starvation (relative risk 3.12, *p* = 0.0090) were identified as risk factors. Diabetes in close relatives, another risk factor for this population (relative risk 2.2, *p* = 0.0131). Abdominal obesity was a risk factor for this population (in females relative risk 2.0, *p* = 0.0010).

**Conclusion:**

The risk factors for type 2 diabetes mellitus described in this rural population include advancing age, diabetes in a first-degree relative and abdominal obesity. This is similar to what has been cited in other studies. At variance, we found that more than 50% of the diabetics reported having suffered malnutrition/starvation in childhood, with more than half of them reporting their dependence on cassava as the only food source during starvation.

## INTRODUCTION

Diabetes mellitus is a chronic disease whose current global spread has the characteristics of a pandemic. Type 2 diabetes defines 85% of the cases, followed by type 1 at 10%. The secondary and gestational types account for about 5%.^1^ The World Health Organisation's report of 2008 on diabetes describes it as a growing epidemic that threatens to overwhelm health services and undermine economies, especially in the developing countries. It currently affects more than 250 million people worldwide, and is expected to affect over 380 million by 2025.^2^ Epidemiological surveys conducted by the Nairobi-based Diabetic Management and Information Center (DMI) give the estimated prevalence of diabetes mellitus in Kenya at 3% in 2003, and above 6% in 2007. In some rural parts of the country such as Nyeri in central Kenya and Kilifi in the coast province the prevalence is as high as 11.6% and above 20% among the richer families in the major urban centers.

Type 2 diabetes mellitus presents with metabolic anomalies: chronic hyperglycaemia, resulting either from the defective secretion or action of insulin (insulin resistance), or both of them combined. The diagnosis of diabetes is made when someone with symptoms of polyuria and polydipsia has venous plasma glucose levels higher than 11.1 mmol/L, or a fasting glycaemia that is higher than 7.0 mmol/L on at least 2 occasions, or glycaemia higher than 11.1mmol/L two hours after a glucose challenge (oral glucose tolerance test).^1^

Studies done in the past (both cross-sectional and longitudinal) have identified a number of risk factors for type 2 diabetes mellitus. The modifiable risk factors include abdominal obesity, excessive alcohol ingestion, poor dietary habits and physical inactivity. The non-modifiable risk factors include ageing and genetic predisposition.^3^

Most studies on diabetes have taken place in Kenya's teaching and national referral hospitals, Moi Teaching and Referral Hospital (MTRH) in Eldoret, and Kenyatta National Hospital (KNH) in Nairobi. These studies have focused mainly on the complications of diabetes. As the Ministry of Health devolves the management, planning and implementation of health policy to the districts, the need for rural-health-facility–based research has become a necessity to guide health policy at the local level.

A rise in the estimated prevalence of diabetes mellitus among rural Kenyans in just four years (2003–2007) has led to the suspicion that there could have been aspects of lifestyle change amongst rural Kenyans that have exposed them to the disease more.

The relationship between lifestyle and the prevalence of diabetes mellitus, and how the changing lifestyles among the Kenyan rural population compares to lifestyles in Western, developed countries, has yet to be determined and documented. If found to be the case, action on risk factors that can be modified through individual, family and community health education and environmental interventions could be instituted. The aim of this study has been to describe risk factors for known type 2 diabetics attending the outpatient diabetes clinic in a rural Kenyan hospital.

## ETHICAL CONSIDERATIONS

The study was approved by both the Moi University School of Medicine Institutional Research and Ethics Committee (IREC) and the AIC Kijabe Hospital research ethics committee.

## METHOD

### Setting

The study was carried out in the outpatient clinics of the AIC Kijabe Hospital, a 170–bed hospital in central Kenya, 60 kilometres west of Nairobi. This is a regional referral hospital.

### Population

This comprised the adult diabetics and non-diabetics who are registered in the ambulatory service of the hospital. They were drawn from this medium-potential agricultural region that is inhabited by mainly subsistence farmers growing maize, beans, bananas and potatoes. Cash crops, grown on a mostly small scale, are tea and coffee. Dairy cattle are reared by zero-grazing for domestic needs.

### Design

This is a cross-section comparative study.

### Sample size determination

The formula used to work out the sample size was borrowed from the textbook *Sampling methods* by William C. Cochrane:

*N* = Z^2^ *p*q / d^2^

Where:

*N* is the population sample

Z is a constant, 1.96, *p* (prevalence) = 3% (0.03), q (1 - *p*) = 0.97,

d = 0.05 (statistically tolerated error)

*N* = 1.96^2^ x 0.03 x 0.97 / 0.05^2^ = 44.72 (45 diabetics and 45 non-diabetics)

### Participant selection

Diabetics were randomly selected from hospital records using a table of random numbers. The non-diabetics, all of whom matched the diabetics for age and gender, were selected from the records of ambulatory patients attending outpatient clinics for follow-up. Their hospital records were selected using a table of random numbers to choose them from their appointment clinic records. The chosen records for both groups were screened to determine whether the demographic details met the inclusion criteria. On the clinic day, further screening was conducted for the non-diabetics by determining their fasting blood sugar levels. Those who had not fasted were requested to return when they had. One record of a fasting blood sugar level below 6.1 mmol/L was used because of the logistical difficulty of persuading them to come back for a second determination. It was observed that most ambulant patients (diabetics and non-diabetic) came to the hospital early in the morning without taking breakfast which made screening convenient for the investigators. The diabetics diagnosed during the study were not included in the study but were counselled and managed.

### Data collection

A structured, pilot-tested questionnaire was used to collect data on demography, estimated monthly family income, past and current nutrition habits, family health history, personal health and social history. A thorough physical examination was carried out by one of the investigators and the findings were documented in the questionnaire. Height was measured using the standard hospital height board for all the subjects. Each subject was asked to remove any form of footwear (shoes, slippers, sandals etc.), headgear, (hat, cap, hair bows, ribbons, turbans, etc). These were removed before the subject could stand on the board, facing the investigator. With the feet together, heels against the board and knees straight, the height was taken and recorded in the questionnaire to the nearest 0.5 cm. Weight was determined using one electronic scale for all the subjects. The scale was connected to the power source and adjusted to read 0.0 kg before use every time.

Weight was taken without shoes or socks and in light clothing. The subjects were asked to step onto the scale with one foot on each side, to stand still with the face forward and arms on the side, and only to step off the scale when asked to. The weight was recorded on the questionnaire to the nearest 0.5 kg.

BMI (Body Mass Index) was calculated for each study subject using the formula: BMI = weight (in kg)/height (in metres) squared.

**TABLE 1:** Characteristics of the 90 participants

Variable	Diabetics	Non-diabetic	*p*-value
	
	*N* = 45	*N* = 45	
**Age (years)**			
Mean	61.8 ± 10.9 (2 SD)	61.4 ± 10.4 (2SD)	
Median	64	62	
Range	(39.00, 82.00)	(35.00, 81.00)	
**Education level**			
a) Less than 8 years	29 (64.44%)	31 (68.89%)	0.655
b) More than 8 years	16 (35.56%)	14 (31.11%)	
**Estimated family monthly income**	7053.3	9411.1	
Median	5000	6000	0.143
Range	(2000.00, 30 000.00)	(1500.00, 35 000.00)	
Males = *n* (%)	13 (28.9%)	13 (28.9%)	
Females = *n* (%)	32 (71.1%)	32 (71.1%)	

**Total (*N*)**	**45 (100%)**	**45 (100%)**	

**TABLE 2:** Current diet and childhood starvation /malnutrition

	Diabetics	Non- diabetics	*p*-value	Relative risk
	
	*N* = 45	*N* = 45		
**Main dietary components**				
Animal protein	2 (4.4%)	9 (20%)		
Unrefined starches + vegetables	43 (95.56%)	36 (80%)	0.0457	0.222 (95% CI, 0.0508 to 0.9718)
**Total**	**45 (100%)**	**45 (100%)**		
**Childhood starvation**				
Yes	25 (55.56%)	12 (26.67%)		
No	20 (44.44%)	33 (73.33%)	0.009	2.083 (95% CI,1.2014 to 3.6128)
**Total**	**45 (100%)**	**45 (100%)**		

**TABLE 3:** Use of cassava as only food during childhood starvation

Cassava use	Diabetics	Non-diabetics	*p*-value	Relative risk (z test = 1.689)
	
	*n* = 25	*n* = 12		
Yes	13 (52%)	2 (16.7%)		
No	12 (48%)	10 (83.3%)	0.091	3.1200 (95% CI, 0.8334 to 11.68)

**Total**	**25**	**12**		

**TABLE 4:** Diabetes and obesity in a first degree relative

	Diabetics	Non-diabetics	*p*-value	Relative risk
	
	*N* = 45	*N* = 45		
**Family diabetes**				
Yes	22 (48.89%)	10 (22.22%)		
No	23 (51.11%)	35 (77.78%)	0.01	2.2 (95% CI, 1.18 to 4.1016)
**Total**	**45 (100%)**	**45 (100%)**		
**Obesity**				
Yes	22 (48.89%)	18 (40%)		1.22 (95% CI, 0.7668 to 1.948)
No	23 (51.11%)	27 (60%)	0.4	
**Total**	**45 (100%)**	**45 (100%)**		

Waist circumference was measured using a constant tension tape, and conducted in a secure room where maximum privacy was ensured. The patient was asked to strip to the undergarments. The measurement was taken at the end of a normal expiration, with the arms relaxed at the sides. The investigator, standing to the side of the patient, marked the inferior margin of the last rib, as well as the superior iliac crest, with a fine pen. The midpoint of these two points was correctly determined with a tape measure and marked with a pen. With the patient's feet together, the tape was applied over the central marked points and the patient asked to wrap it round themselves. After confirming that the tape was horizontal across the back and the front and that the patient was relaxed and breathing gently, the circumference was read at the level of the tape to the nearest 0.1 cm and recorded on the space provided in the questionnaire.

Every subject was interviewed and examined in one day and any newly diagnosed conditions were managed and a structured referral and/or follow-up was established.

### Data handling and analysis

Data from the questionnaires were double-checked and cleaned before it was entered into a computer by one of the investigators. The Kruskal–Wallis test was used to compare the medians for the continuous variables, while the chi-squared test was used for the categorical variables. The z-test was used to calculate the relative risk for all identified risk factors.

## RESULTS

The study was conducted between 30 May 2006 and 24 April 2007. Complete data was available for all the 90 participants.

The educational level of the diabetics and non-diabetics groups is the same. The estimated monthly income for the diabetics is lower than that of the non-diabetics but the difference is not statistically significant.

Both diabetics and non-diabetics live on a diet of mainly unrefined starches and vegetables, but the non-diabetics have a higher percentage with ingestion of animal protein. Childhood starvation and starvation were found to predispose one to a relative risk of type 2 diabetes mellitus in this study population.

The results showed that use of cassava for sustenance during the period of childhood starvation tripled the risk of developing type 2 diabetes mellitus.

Family history of diabetes in a first degree relative presents a risk that is more than double in this population, while obesity in a first degree relative has a relative risk of 1.22, but it is not statistically significant (*p* = 0.3988).

Abdominal obesity presents a double risk of developing diabetes among the females in this population. The male risk in abdominal obesity is three times, but the risk is not statistically significant (*p* = 0.1250).

## DISCUSSION

This study was carried out in a rural health facility in Kenya to compare type 2 diabetics with non-diabetics who were matched for age and gender. The aim was to identify the characteristics that were different in diabetics as compared to non-diabetics and whether these could be risk factors for the disease. The study was driven by the apparent rapid rise in the prevalence of type 2 diabetes mellitus among rural Kenyans–an increase of 3% to 7% in four years according to population surveys conducted by the Diabetes Management and Information Center (DMI) based in Nairobi, Kenya.

**TABLE 5:** Abdominal obesity

Abdominal obesity	Diabetic	Non-diabetics	*p*-value	Relative risk
	
	*N* = 45	*N* = 45		
**Females**				
Yes	28 (87.5%)	14 (43.75%)		2.0 (95% CI, 1.3218 to 3.0261)
No	4 (12.5%)	18 (56.25%)	0.001	
**Total**	**32**	**32**		
**Males**				
Yes	6 (46.15%)	2 (15.35%)		3.0 (95% CI, 0.7371 to 12.2093)
No	7 (53.85%)	11 (84.65%)	0.1250	
**Total**	**13 (100%)**	**13 (100%)**		

In this rural population (mainly the Kikuyu) in central Kenya, the diabetics attending the outpatient clinic were of average age above sixty years. A factor that contributed to significantly higher incidences of type 2 diabetes, compared to non-diabetics of the same age and gender, included a significant period of childhood starvation. Intrauterine growth retardation in children born small for gestational age, remaining short, and being characterised by insulin resistance, are at risk for type 2 diabetes mellitus.^4^ Birth weights, though contemplated in the study, were not possible to establish for this population that were elderly and mostly born at home. The Leningrad study, which studied the effect of starvation *in utero* and infancy, is the only literature we were able to find that investigated the relationship between childhood starvation and type 2 diabetes mellitus.^6^ The Thrifty genotype theory of the 1960s, which hypothesised about the presence of a starvation selected gene–efficient in energy storage but detrimental in affluent times–may need further study/research.^5^

In contrast is maturity-onset diabetes of the young (MODY), in which chronic hyperglycemia starts in childhood or adolescence and is associated with obesity and a strong family history of type 2 diabetes.

Ingestion of cassava as the main food for sustenance during the period of childhood starvation presented a threefold risk of developing type 2 diabetes in this population. Ingestion of cassava in the tropics has been associated with a nonalcoholic form of chronic pancreatitis. This differs from that found in the temperate zone in that it occurs in youth, has a more accelerated course, a higher prevalence of pancreatic calculi and diabetes, and has a tendency to cause pancreatic malignancy. The diabetes is severe and requires insulin, although it is non-ketotic. Most cases have been reported in the Indian subcontinent.^7^ The etiology of the tropical pancreatitis remains largely unclear, although cyanogens toxicity, attributed to poorly prepared cassava, antioxidant deficiency (in malnutrition), and possibility of genetic predisposition were all entertained as possible contributing factors. None of the participants in our study had features of chronic pancreatitis (abdominal pain and steatorrhoea).

Abdominal obesity presented a twofold risk of developing type 2 diabetes in our female participants. The parameter was determined by the waist circumference whose upper limit was 88 centimetres in females. Although general obesity (determined by body weight in kilogram/height in metres squared) has been reported as a major risk factor for type 2 diabetes mellitus, intra-abdominal adipose accumulation has been found to be the key correlate to the metabolic syndrome.^8–11^ The metabolic syndrome is a cluster of metabolic abnormalities that have been observed to predispose one to cardiovascular disease and type 2 diabetes. These include individuals being atherogenic, prothrombotic, hypertensive; they had dyslipidemia; inflammatory metabolic changes with impaired glucose tolerance.^11^

Diabetes reported in a first-degree relative presented a twofold risk of type 2 diabetes in our study population. Diabetes type 2 in siblings and members of the nuclear family has been reported in previous studies.^12–14^ Family history of type 2 diabetes is not only a risk factor for the disease but could be used positively for risk awareness and risk-reducing behaviour. It can also provide a useful tool for the screening and possible delay, if not prevention, of the disease.

Dietary and social habits (smoking cigarettes and the excess use of alcohol) were studied. There was no significant difference between the reported dietary habits of the diabetics compared to the non-diabetics. The majority reported eating food that was mainly fresh and grown on their small parcels of land. The main components of their diet were predominantly unrefined starches and green vegetables. The diabetics reported having depended on the same diet prior to the diagnosis of their condition and the only difference was in the quantities of starch consumed now (less starches). The use of alcohol and cigarette smoking were not significantly reported. Studies conducted in other centres have reported a significant association of diet and lifestyle as risk factors for type 2 diabetes mellitus. A diet composed of high quantities of animal protein and refined starches has been associated with increased risk for type 2 diabetes mellitus.^15–17^ The level of physical activity was not determined for this population. Physical inactivity is a risk factor for type 2 diabetes mellitus and has been reported in other studies.^17^

### Limitations of the study

The findings of this study cannot be generalised to the rest of the country due to differences in genetic make-up and the socioeconomic and dietary habits in different rural communities in Kenya, which has 42 ethnic groups.

This was a hospital-based study and results may not necessarily concur with those of a population study in the same community under other conditions. AIC Kijabe hospital charges much more than those run by the Ministry of Health and does not serve the very poor in the community who may not be able to afford the services rendered in this hospital.

The information received during the interview depended mostly on recall. Recall bias is known to affect the accuracy of data and contribute to systemic error. To ensure the smooth flow of information and minimise this bias, the interview was not conducted in a question and answer format but in a discussion format, in which responses to questions in the questionnaire were noted without interrupting the flow of the conversation.

## CONCLUSION

The risk factors in this study that have been cited in other studies include advancing age, diabetes in a first-degree relative and central obesity. Those at variance with the findings of other studies included: starvation/malnutrition in childhood and use of cassava during that period, and a diet consisting, for the most part, of unrefined starches and greens, even prior to diagnosis of the condition.
